# Axillary skin crease incision versus conventional posterolateral incision in open repair of patent ductus arteriosus for extremely low birth weight infants: a retrospective study

**DOI:** 10.1186/s12893-023-02081-9

**Published:** 2023-06-23

**Authors:** Mitsumasa Okamoto, Yudai Tsuruno, Hiroaki Fukuzawa

**Affiliations:** Department of Pediatric Surgery, Himeji Red Cross Hospital, 1-12-1, Shimoteno, Himeji, Hyogo 670-8540 Japan

**Keywords:** Axillary skin crease incision (ASCI), Extremely low birth weight (ELBW) infants, Open patent ductus arteriosus (PDA) repair, Posterolateral incision (PLI), Limited clipping angle

## Abstract

**Background:**

Thoracotomy with posterolateral incision (PLI) is commonly used for surgical repair of patent ductus arteriosus (PDA) in extremely low birth weight (ELBW) infants. Some reports have described thoracotomy for PDA using an axillary skin crease incision (ASCI) in consideration of cosmetic problems such as surgical wounds and thoracic deformities, but the details remain unclear.

**Methods:**

In this study, we performed clipping ligation by thoracotomy with ASCI for ELBW infants with PDA from 2011 to 2015 for the purpose of improving cosmetic results, and retrospectively compared the results with those for conventional PLI cases performed from 2016 to 2020.

**Results:**

ASCI was found to be associated with serious surgical complications and showed a significant difference in outcome parameters only for surgery time, suggesting a safety problem for ASCI. Considering these results, PLI allows clipping of the nearby PDA from the thoracotomy wound while looking straight ahead, whereas the PDA in ASCI is positioned deep and oblique to the thoracotomy wound, so the clipping angle is limited and accurate completion of the procedure is difficult.

**Conclusions:**

Regarding PDA repair in ELBW infants, ASCI shows a high risk of serious surgical complications. Conventional PLI remains preferable for safe and accurate results.

## Background

Repair of patent ductus arteriosus (PDA) in neonates and infants is usually performed by clipping ligation under thoracotomy through posterolateral incision (PLI), bringing good results [1–3]. Some reports have described thoracotomy for various pediatric surgeries using axillary skin crease incision (ASCI) in consideration of cosmetic problems such as surgical wounds and thoracic deformity [4–6]. Such reports have described successful application of ASCI to PDA ligation under thoracotomy, but the details are not clear [4, 5]. In this study, we performed clipping by ASCI on extremely low birth weight (ELBW) infants with PDA for the purpose of improving cosmetic aspects, and retrospectively compared the results with those for cases of conventional PLI to clarify the safety of these surgical procedures.

## Materials and methods

This study was undertaken with the approval of the Ethics Committee for Clinical Research at Himeji Red Cross Hospital, Hyogo, Japan (registration no. 2022–31). Informed consent was obtained from the guardian of each patient prior to surgery.

**Fig.1:**
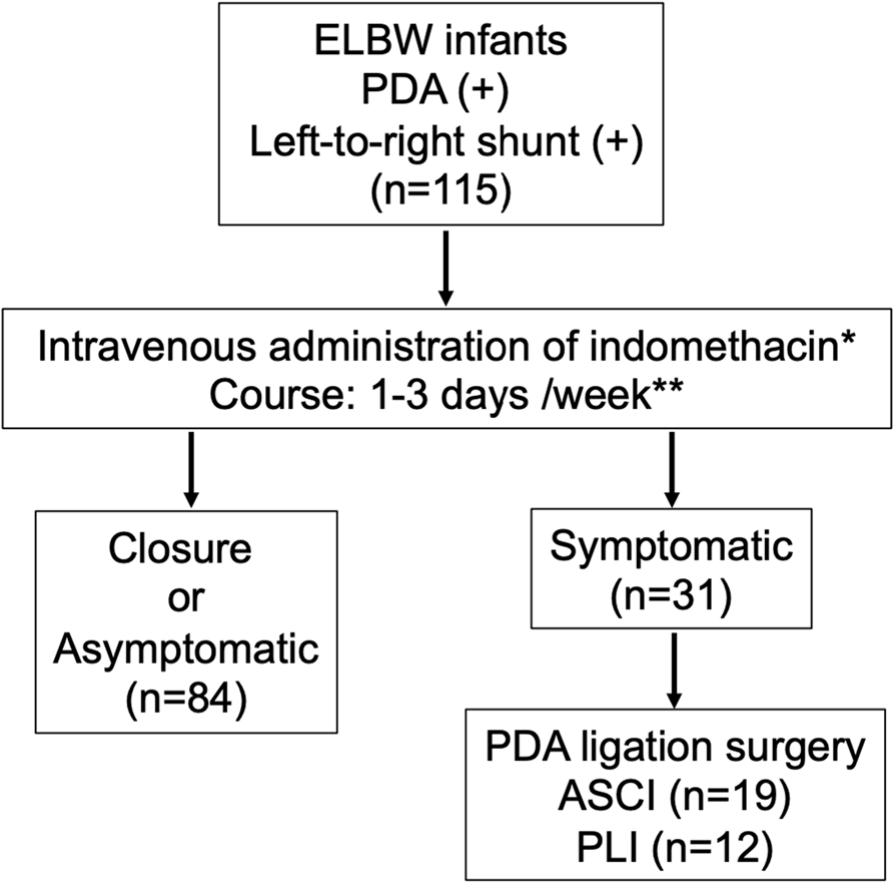
Treatment protocol for PDA of ELBW infants. * 0.2 mg/kg, followed by 0.1 mg/kg or 0.2 mg/kg depending on gestational age and symptoms, ** Number of courses: maximum of 4. *ELBW* extremely low birth weight*, PDA* patent ductus arteriosus, *ASCI* axillary skin crease incision, *PLI* posterolateral incision

From 2011 to 2020, a total of 31 ELBW (birth weight < 1000 g) infants with PDA who became symptomatic after failure of indomethacin administration underwent clipping ligation by thoracotomy. Nineteen cases underwent clipping surgery by ASCI between 2011 and 2015 and the remaining 12 underwent PLI between 2016 and 2020 (Fig. 1).

PLI was started at the mid-axillary line and ran close to the tip of the scapula. The latissimus dorsi muscle was retracted and the serratus anterior muscle was incised at the superior margin of the 4th rib to allow 3rd intercostal thoracotomy. ASCI was made on the skin crease running between the anterior and posterior axillary folds. The pectoralis major and latissimus dorsi muscles were retracted and 4th intercostal thoracotomy was performed. With each approach, the lung was retracted to expose the aorta, PDA and recurrent laryngeal nerve. Pleura between cephalad and the distal corner of the PDA was opened along the aorta lengthwise to avoid damage to the recurrent laryngeal nerve. A titanium clip was then applied on the side close to the aorta. All data are expressed as median and interquartile range (IQR). Demographic factors, clinical findings and blood test data at surgery were compared between ASCI and PLI using the Mann–Whitney test or chi-squared test using SPSS II Statistics software (SPSS Inc.). Significance was defined as a value of *P* < 0.05.

## Results

Among the 31 cases, no complications were encountered in any of the 12 PLI cases (0%), whereas 6 of the 19 cases of ASCI (32%) developed serious surgical complications (3 cases of post-clipping ductal bleeding requiring transfusion, 3 cases of left vocal cord paralysis) (chi-square test; *P* = 0.037; Table 1).

**Table 1:** Comparison of surgical complication rates between ASCI and PLI

ASCI (*n *= 19)	PLI (*n* = 12)	*P* value
6/19 (32%)	0/12 (0%)	0.037*
Post-clipping ductal bleeding (*n* = 3)		
Left vocal cord paralysis (*n* = 3)		

*ASCI* Axillary skin crease incision, *PLI* Posterolateral incision

^*^Statistically significant, *P* < 0.05

Twelve PLI cases were all alive, but 3 ASCI cases with post-clipping ductal bleeding died of hemorrhagic shock, intraventricular hemorrhage, and multiple organ failure on days 1, 1, and 17 after surgery, respectively (chi-square test; *P* = 0.22; Table 2).

**Table 2:** Comparison of postoperative mortality rates between ASCI and PLI

ASCI (*n* = 19)	PLI (*n* = 12)	*P* value
3/19 (16%)	0/12 (0%)	0.22
Hemorrhagic shock (*n* = 1)		
Intraventricular bleeding (*n* = 1)		
Multiorgan failure (*n* = 1)		

*ASCI* Axillary skin crease incision, *PLI* Posterolateral incision

To investigate the high incidence of severe surgical complications in ASCI, the patient’s characteristics, blood test data at surgery and surgical outcomes were compared between ASCI and PLI. Only median surgery time differed significantly between groups, being significantly longer in ASCI (62 min, IQR 54–76 min) than in PLI (30 min, IQR 27–33 min; *P* < 0.001) (Table 3, 4).

**Table 3:** Comparison of the patient’s characteristics and blood test data between ASCI and PLI

	ASCI (*n* = 19)	PLI (*n* = 12)	*P* value
Gestational age (weeks)	24.3 (23.3–25.3)	25.3 (24.0–26.2)	0.43
Birth weight (g)	630 (544–796)	726 (565–907)	0.22
Weight at surgery (g)	767 (558–906)	956 (793–1021)	0.15
Age at surgery (days)	20 (15–32)	28 (23–36)	0.18
Indomethacin administration period (days)	6 (5–8)	8 (7–9)	0.46
Congenital anomalies (%)	0	0	1
White blood cell (× 10^2^/μl)	108 (88–149)	113 (95–130)	0.79
Red blood cell (× 10^4^/μl)	321 (296–339)	327 (316–378)	0.27
Hemoglobin (g/dl)	10.5 (9.5–11.5)	11.4 (10.8–12.3)	0.12
Platelet (× 10^4^/mm^3^)	31.2 (25.4–35.2)	35.9 (28.4–40.5)	0.18
PT (sec)	14.2 (13.0–15.1)	13.3 (11.4–14.6)	0.13
CRP (mg/dl)	0.00 (0.00–0.01)	0.02 (0.00–0.18)	0.20

*ASCI* Axillary skin crease incision, *PLI* Posterolateral incision, *PDA* Patent ductus arteriosus, *PT* Prothrombin time, *CRP* C-reactive protein

**Table 4:** Comparison of surgical outcomes between ASCI and PLI

	ASCI (*n* = 19)	PLI (*n* = 12)	*P* value
Diameter of PDA (mm)	3.5 (3.0–4.5)	4.0 (4.0–4.5)	0.69
Surgery time (min)	62 (54–76)	30 (27–33)	< 0.001*
Length of hospital stay (days)	125(109–144)	124 (116–135)	0.98

*ASCI* Axillary skin crease incision, *PLI* Posterolateral incision, *PDA* Patent ductus arteriosus

^*^Statistically significant, *P* < 0.05

After excluding the 6 cases of ASCI with surgical complications, the remaining 13 cases were compared with the 12 cases of PLI to exclude the influence of surgical complications. Median surgery time was still significantly longer for ASCI without surgical complications (62 min, IQR 46–73 min) than for PLI (30 min, IQR 27–33 min; *P* = 0.001) (Table 5,6).

**Table 5:** Comparison of the patient’s characteristics and blood test data between ASCI without surgical complications and PLI

	ASCI without surgical complications (*n* = 13)	PLI (*n* = 12)	*P* value
Gestational age (weeks)	24.7 (23.8–26.1)	25.3 (24.0–26.2)	0.94
Birth weight (g)	642 (560–810)	726 (565–907)	0.25
Weight at surgery (g)	797 (632–974)	956 (793–1021)	0.17
Age at surgery (days)	20 18–30)	28 (23–36)	0.18
Indomethacin administration period (days)	6 (5–8)	8 (7–9)	0.34
Congenital anomalies (%)	0	0	1
White blood cell (× 10^2^/μl)	101 (85–149)	113 (95–130)	0.36
Red blood cell (× 10^4^/μl)	321 (276–336)	327 (316–378)	0.12
Hemoglobin (g/dl)	10.5 (9.5–11.5)	11.4 (10.8–12.3)	0.13
Platelet (× 10^4^/mm^3^)	31.2 (27.0–41.0)	35.9 (28.4–40.5)	0.56
PT (sec)	14.0 (13.0–15.0)	13.3 (11.4–14.6)	0.14
CRP (mg/dl)	0.00 (0.00–0.07)	0.02 (0.00–0.18)	0.51

*ASCI* Axillary skin crease incision, *PLI* Posterolateral incision, *PDA* Patent ductus arteriosus, *PT* Prothrombin time, *CRP* C-reactive protein

**Table 6:** Comparison of surgical outcomes between ASCI without surgical complications and PLI

	ASCI without surgical complications (*n* = 13)	PLI (*n* = 12)	*P* value
Diameter of PDA (mm)	4.0 (3.0–4.8)	4.0 (4.0–4.5)	0.55
Surgery time (min)	62 (46–73)	30 (27–33)	< 0.001*
Length of hospital stay (days)	129 (118–153)	124 (116–135)	0.38

*ASCI* Axillary skin crease incision, *PLI* Posterolateral incision, *PDA* Patent ductus arteriosus

^*^Statistically significant, *P* < 0.05

These findings clarified that the markedly higher incidence of surgical complications and long surgery time were attributable to the ASCI procedure itself.

## Discussion

Surgical treatment for PDA in ELBW infants is performed when medical management cannot stabilize symptoms due to a hemodynamically severe defect [7]. Video-assisted thoracoscopic surgery (VATS) PDA ligation was first reported in 1993 [8] and has recently been performed as a minimally invasive surgical technique for PDA closure, including in ELBW infants [9]. Clipping under thoracotomy by PLI is also commonly used and provides good outcomes [1–3]. As described above, our 12 PLI cases displayed no problems associated with surgery. There seems to be little evidence to suggest better wound status and cosmesis in infants following VATS PDA ligation, but VATS can offer potential reduction of cosmetic problems by avoiding rib spreading and division of muscles [10, 11].

It has been reported that ASCI has been applied to open surgeries for congenital esophageal atresia and congenital pulmonary airway malformation, and that excellent results have been obtained in terms of cosmetic appearance [4–6]. Although thoracotomy for PDA using an ASCI to address cosmetic problems such as surgical wounds and thoracic deformity, the number of such cases is small and detailed data on patient demographics and clinical outcomes have been scarce [4, 5]. To improve the cosmetic outcomes, we performed open PDA clipping ligation by ASCI in 19 ELBW infants, but review of the statistical data revealed clear safety issues (Table1, 2, 4, 6).

**Fig. 2:**
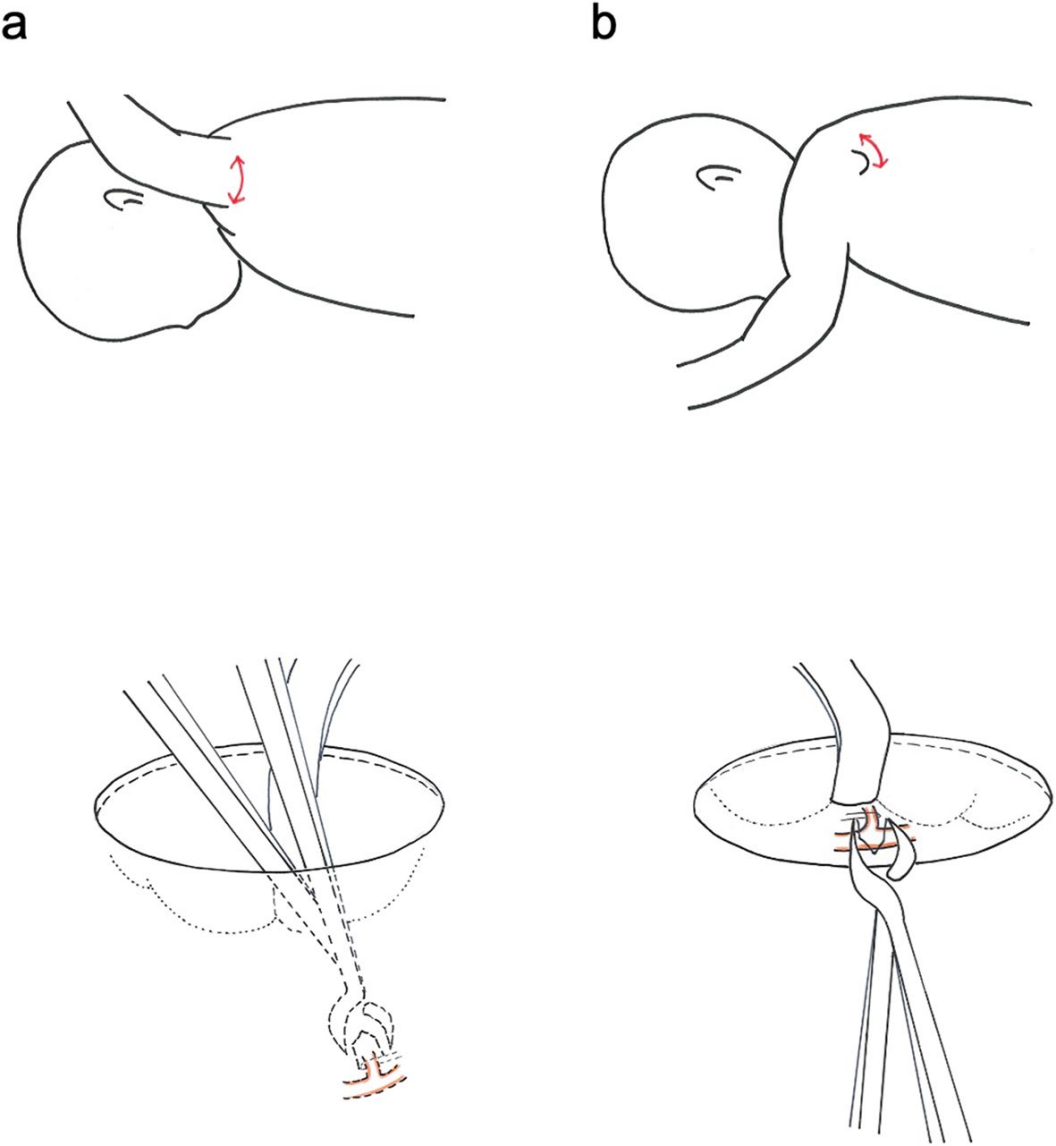
Surgical schema for open patent ductus arteriosus (PDA) repair with axillary skin crease incision (ASCI) and posterolateral incision (PLI). PDA was located deep and obliquely from the thoracotomy wound, severely limiting the angle of clipping by ASCI (**a**). In PLI, the PDA could be seen directly in front of and near the thoracotomy wound (**b**)

In considering potential causes, we found that the PDA with the ASCI approach was located deep and obliquely from the thoracotomy wound, severely limiting the angle of clipping, requiring more time for surgery and reducing the accuracy of maneuvers (Fig. 2a). On the other hand, with the PLI approach, the PDA could be seen directly in front of and close to the thoracotomy wound, so maneuvers could be performed quickly and accurately (Fig. 2b).

Bleeding is considered rare in both open and thoracoscopic repair of PDA [1, 12], but reports have described serious complications such as inadvertent ligation of the left pulmonary artery [13]. Among infants with low birth weight, the incidence of left vocal cord paralysis due to open PDA surgery is relatively high, as are the incidences of comorbidities such as gastroesophageal reflux disease [14]. The “safety” is thus a particularly important issue in PDA surgery in ELBW infants.

## Conclusions

ASCI is a useful procedure for improving the cosmetic appearance in thoracotomy, but in PDA clipping for ELBW infants this approach may develop serious surgical complications due to the limited clipping angle. We emphasize that in cases of open surgery, PDA repair should be performed safely and accurately using the PLI.

## Data Availability

The datasets of the current study are available from the corresponding author upon reasonable request.

## Abbreviations

PDA: Patent ductus arteriosus
PLI: Posterolateral incision
ASCI: Axillary skin crease incision
ELBW: Extremely low birth weight
IQR: Interquartile range
PT: Prothrombin time
CRP: C-reactive protein
VATS: Video-assisted thoracoscopic surgery
